# Exploring Mental Health and Academic Outcomes of Children Receiving Non-manualized, Transdiagnostic, Task-Shifted Mental Health Care From Their Teachers in a Low-and-Middle Income Country

**DOI:** 10.3389/fped.2022.807178

**Published:** 2022-03-21

**Authors:** Juliana L. Vanderburg, Choden Dukpa, Abhishek K. Rauniyar, Priscilla Giri, Surekha Bhattarai, Arpana Thapa, Bradley N. Gaynes, Karen Hampanda, Molly M. Lamb, Michael Matergia, Christina M. Cruz

**Affiliations:** ^1^School Psychology Program, School of Education, University of North Carolina at Chapel Hill, Chapel Hill, NC, United States; ^2^Darjeeling Ladenla Road Prerna, Darjeeling, India; ^3^Department of Epidemiology, Colorado School of Public Health, Aurora, CO, United States; ^4^Department of Psychiatry, School of Medicine, University of North Carolina at Chapel Hill, Chapel Hill, NC, United States; ^5^Department of Epidemiology, Gillings School of Global Public Health, University of North Carolina at Chapel Hill, Chapel Hill, NC, United States; ^6^Center for Global Health, Colorado School of Public Health, Aurora, CO, United States; ^7^Department of Obstetrics and Gynecology, University of Colorado Anschutz Medical, Aurora, CO, United States; ^8^Broadleaf Health and Education Alliance, Stroudsburg, PA, United States

**Keywords:** child mental health, task-shifting, low-and-middle income countries (LMIC), school-based intervention, teachers

## Abstract

A majority of children worldwide who face mental health difficulties, especially in low-and-middle income countries, remain undiagnosed and untreated. This deficit roots in part from a lack of trained professionals qualified to provide care. Task-shifting the provision of treatment to teachers, individuals with consistent access to children, can reduce the care gap. The current study investigated whether the implementation of a pilot trial of *Tealeaf-Mansik Swastha* (Teachers Leading the Frontlines—Mental Health; “Tealeaf”) was associated with improvements in child mental health and academic outcomes. Tealeaf is a transdiagnostic, non-manualized, task-shifting intervention in which teachers identify students in need of mental health care and then provide task-shifted care for them using an emerging, novel therapy modality, “education as mental health therapy” (Ed-MH). Pre-post standardized quantitative measures focused on child mental health status and academics. The measures were completed by multiple raters and compared to determine whether changes occurred. Results indicated that primary teacher raters observed significant improvements in child mental health symptoms overall, while secondary teacher raters and caregivers noted improvement for certain diagnostic categories. Caregivers observed on average a decreased impact of their children's mental health symptoms on their children's lives. Academically, math scores significantly improved while reading trended toward significance. Preliminary evidence overall supports the viability of Tealeaf and Ed-MH for positively impacting child mental health and academics. Future directions include the implementation of a formalized, randomized-controlled trial to strengthen preliminary outcomes.

## Introduction

Globally, the mental and behavioral health needs of children often remain unaddressed. The importance of these concerns has been well-documented; mental health difficulties may impact healthy development, academics, quality of life, and well-being in childhood, and predict poor health, unemployment, and contact with law enforcement agencies in adulthood (1). Despite the pervasive impact of childhood mental health difficulties, the majority of children worldwide with a diagnosable and treatable mental health condition do not receive appropriate, indicated levels of care, defined as care aiming to reduce or eliminate symptoms of a diagnosable mental health disorder (2). In low-and-middle income countries (LMICs), these numbers are even more dire; for instance, in India, the site of the current study, only about 5,00,000 of over 50 million children with mental health concerns receive care, creating a significant service gap (3). This service gap exists in part due to a lack of qualified, trained professionals available to provide evidence-based mental health care (3, 4).

Innovative care systems that utilize alternative human resources are needed to address the service gap. Task-shifting has been proposed as a method of increasing the number of professionals available to provide care (5). In a task-shifted system of care, experienced professionals provide lay community members with the training and knowledge needed to implement specialized public and mental health interventions (2). The efficacy of this approach has been repeatedly validated for adults and adolescents (5–7), while early evidence has supported the potential for task-shifted, indicated care to improve child mental health outcomes as well, specifically in schools (8).

Schools have been proposed as a viable locale for the implementation of mental health interventions delivering indicated care (9). School professionals have consistent access to children, along with a strong understanding of child development principles and education, facilitating their ability to learn and deliver mental and behavioral health interventions to improve child quality of life (9). Indicated care delivery in schools has been limited to school counselors or outside professionals (9, 10). Many under-resourced schools, however, do not have counselors or outside professionals who can deliver care in schools; this lack of available human resources is thus a significant barrier to providing indicated care in schools (8, 11). Further, systemic challenges, such as (1) quality of therapy training, (2) a lack of time for school professionals to deliver the intervention, (3) coordinating mental health care with academic scheduling, (4) community stigma, (5) contradicting priorities of health and education systems, and (6) a lack of support from families, also influence the successful implementation of school-based mental health interventions (12).

Given the potential of schools for decreasing the mental health service gap, innovative, flexible approaches are needed to address these barriers. A transdiagnostic, teacher-led, task-shifted model of indicated child mental health care delivery may alleviate many of the challenges described above (9). Ubiquitous in schools, teachers in LMICs are uniquely positioned to deliver task-shifted children's mental health care given their consistent access to children and may be feasibly leveraged for task-shifted intervention, addressing human resource barriers in LMIC contexts (9). Teachers have been shown to be able to deliver manualized, diagnosis-specific care to improve child mental health symptoms (8, 10, 13, 14). However, due to time constraints, at scale, teachers may be unable to learn and deliver such structured, manualized care on top of their teaching duties (15, 16). A transdiagnostic approach to care may better align with teachers' available time to deliver care, potentially addressing logistical barriers (17). Transdiagnostic care applies “the same underlying principles across mental disorders, without tailoring the protocol to specific diagnoses” (17), thus allowing teachers to efficiently learn one set of therapeutic skills for all diagnoses. Teachers can then deliver care to any child with any mental health challenge. Further, teachers have reported that delivering care in a form that is flexible, non-manualized, and allows for adaptation, as opposed to strict, manualized care, is critical to their potential sustainment in delivering task-shifted care (18). This flexibility could potentially address barriers related to intervention acceptability and sustainability (18). Only one study, from this group of authors, has examined whether the teacher-led delivery of transdiagnostic, non-manualized care is associated with improved child mental health outcomes, showing preliminary evidence of improvement (19).

Moreover, children's mental health struggles contribute to falling behind academically and addressing their mental health can lead to improved academic outcomes (20, 21). Thus, teachers delivering care may synergistically influence, at least in part, children's ability to obtain academic skills. No studies have examined the academic outcomes of children receiving task-shifted mental health care from their teachers.

*Tealeaf-Mansik Swastha* (Teachers Leading the Frontlines—Mental Health; “Tealeaf”) is a task-shifted, transdiagnostic, non-manualized intervention in which teachers deliver indicated mental health care to targeted children in their classrooms displaying clinical-level symptoms of mental health concerns. Teachers attend a brief training on the basics of child mental health and learn evidence-based behavioral and therapeutic techniques. With the support of qualified supervisors, teachers select students displaying clinical-level mental health concerns and then deliver evidence-based intervention throughout the school year. A pilot trial of Tealeaf in 2018 illustrated the willingness to serve as lay counselors (18), the ability of teachers to accurately select students for intervention following the training (22), and the ability of teachers to deliver task-shifted care feasibly and with fidelity (19). Child mental health symptom outcomes as reported by teachers were explored with a small sample size, and findings indicated a signal of improvement. Child mental health outcomes as reported by parents and academic outcomes have yet to be explicitly examined. The current study is situated within a pilot trial that studied teacher, caregiver, and student acceptability of teachers delivering Tealeaf to students as the primary outcome. Here, we explore secondary outcomes of the trial, child mental health as reported by teachers and by parents and academic outcomes. We hypothesized that child participants in Tealeaf would experience significant improvements in both mental health and educational domains as a potential signal of efficacy.

## Methods

### Study Setting

The “Tealeaf” intervention took place in 2019 within five primary schools situated in the Darjeeling Himalayas, a region of the State of West Bengal in India. Darjeeling is home to a population of around 8,00,000 individuals from a diversity of ethnic and cultural backgrounds, including those of Nepali descent and members of indigenous groups, minorities in Bengali-dominated West Bengal (23). Economic conditions in Darjeeling are poor; most residents are tea plantation laborers who earned daily wages of 176 Indian rupees (INR; $2.40) at the time of the pilot trials in 2018 and 2019 and now earn an average of 202 INR daily [$2.76; (24)]. Despite these conditions, many families in Darjeeling opt to send their children to low-cost private (LCP) schools that charge a modest rate of tuition, perceiving this option to yield a higher quality, English education (25). The poor economic conditions in Darjeeling, in conjunction with the emphasis on education, has led to a recent flood of out-migration to urban centers among many young people in the region as a result of their rising social and educational aspirations (26). Prevalence rates of child mental health concerns in Darjeeling have not been published; in similar rural regions of West Bengal, however, pediatric mental health difficulties such as clinically elevated rates of anxiety, hyperactivity, and non-compliance are estimated to be at around 33.3% (27). Unpublished observations from a needs assessment conducted by the authors in 2017 indicated that four mental health professionals addressed the needs of ~1,00,000 youth in Darjeeling at that time.

### Participants

Teachers (*n* = 19), students (*n* = 26), and caregivers (*n* = 29) from 9 rural LCP schools in Darjeeling participated in the overall program acceptability pilot trial. Schools were considered for study inclusion if they employed at least 3 teachers; did not receive government aid; charged an annual fee of 1,15,000 INR ($180 USD); and served families with a daily average income of <725 INR ($10 USD). These criteria were selected for the purpose of reaching children in Darjeeling with poor access to care and were based on an earlier study in Darjeeling targeting the same population of children (25). The study coordinator (PG) identified schools meeting inclusion criteria based on prior community knowledge and field visits. She and other members of the Darjeeling-based team then communicated with school administrators regarding the intervention. If the school leadership agreed, the team approached teachers in the schools. All teachers in the 9 LCP schools were approached for the trial unless they (1) had no prior experience of teaching primary grade levels for at least 1 year; (2) were aged 18 years or below; or (3) held a prior conviction or were under active investigation for child-related misconduct. All participants completed informed consent. Caregivers completed consent for their children to be considered for targeted intervention, then gave additional consent if their child was selected for intervention. Children over age 7 were verbally assented. Of the 19 teachers who were consented into the study, thirteen teachers completed all study activities and pre-post-quantitative measures. Of the 29 caregivers whose children were targeted for intervention, 25 completed pre- and post-quantitative measures.

### Procedures

Teachers were recruited and enrolled in Tealeaf between December 2018 and January 2019. Teachers completed the 10-day Tealeaf training on child mental health and behavioral intervention in February 2019. The training, led by a Darjeeling-based psychiatric social worker, included psychoeducation on anxiety, disruptive, and mood disorders as well as learning to (1) identify children with clinical level mental health struggles, (2) perform basic functional behavior assessments, and (3) use transdiagnostic therapeutic techniques. Table 1 breaks down Tealeaf into components. Rather than providing training on specific Diagnostic and Statistical Manual (DSM)-5 diagnoses (28), Tealeaf uses a framework for conceptualizing behavior as fitting into broad categories of anxiety, disruptive, or mood to technically level mental health theory and therapy for teachers lacking prior psychiatric training (22).

**Table 1:** Core intervention components.

**Intervention component**	**Activity**	**Description**
Professional development and regular supervision	Training	PD: 10-day interactive training for teachers in identification of children with mental health needs, basic functional behavioral assessments and tenets of CBPT, particularly behavior activation, play, and cognitive restructuring Supervision: Monthly discussion with and/or observation of the teacher working with the child to provide concrete guidance and technique Case reviews: Monthly case reviews are conducted with a team of local and international mental health experts.
Assessment	Behavior analysis	Observations of targeted students through a behavioral lens for key behaviors using the AABC Chart and the Themes of the AABC Chart. Supplemented by collateral from caregivers for further observations from student's home lives.
Tailored instruction; therapeutic interactions and skills practice	Behavior plan	A behavior plan (4Cs) incorporating CBPT and classroom-based therapeutic techniques, that is, Ed-MH, that target school-specific behaviors (1:1 and during instructional time), to be used daily. Use of plan in student home is highly encouraged.
Therapeutic interactions and skills practice	1:1 student interaction	Per behavior plan students engage in 1:1 interactions with teacher during or outside of class. These interactions include Ed-MH, CBPT and relationship-building activities.
Therapeutic interactions and skills practice	1:1 family interaction	With support and guidance from teachers, primary caregivers have roles in behavior analysis, implementation of behavior plans, development of positive parental relationships, and reinforcement of positive behaviors.

*PD, professional development; CBPT, Cognitive Behavior Play Therapy; AABC Chart, Activating Event, Automatic Thought and/or Feeling, Behavior, and Consequence Chart; 4Cs, Cause, Change, Connect, Cultivate; Ed-MH, education as mental health therapy*.

Following the training, teachers returned to their schools and selected two students each from their classrooms for targeted intervention, with support from supervising psychiatric social workers (“supervisors”) based in Darjeeling who were study staff and have expertise in child mental health. Teachers based the selections on their observations of child classroom behaviors and according to perceptions of their students' needs for mental health support. Tealeaf-trained teachers have been shown to select students for intervention with moderate accuracy (22).

After selecting the children, teachers worked with supervisors to analyze selected children's behaviors through basic functional behavior assessments using study-specific tools leveling this task, the AABC Chart (Antecedent, Automatic Thoughts/Feelings, Behaviors, and Consequences; “AABC Chart”) and the Themes of the AABC Chart (Supplementary Figure 1). Based on their observations of needs from the AABC Chart, they then created unique, flexible intervention plans for students (“Cause, Change, Connect, Cultivate”; “4Cs”; Supplementary Figure 1) using a “menu” of evidence-based techniques derived from the training (Supplementary Figure 2). The therapeutic techniques were grounded in Cognitive Behavior Play Therapy (CBPT) and designed to be deliverable within the workflow of teaching and classroom activities or during one-on-one brief sessions, at the teachers' discretion (29).

The “dose” of care is each therapeutic interaction between the teacher and student, whether in the classroom or in one-on-one brief sessions, deviating from traditional lay counselor interventions that are modeled on office-based one-on-one therapy sessions. To illustrate such a dose, we provide the example of a teacher working with a depressed child who has difficulty completing classwork due to anhedonia and has automatic thoughts of being incapable of completing classwork. To counter these automatic thoughts, the teacher may lessen the child's required classwork as a behavioral activation measure, enabling the child to complete classwork that, in turn, is evidence against the child's automatic thought of being incapable of completing classwork. The teacher can then increase expected classwork over time as a form of exposure until the student can complete all expected work. Teachers' use of academically contextualized therapeutic techniques, as in the example, indicates the potential emergence of a new form of therapy, “education as mental health therapy” (Ed-MH), explored in a separate manuscript from this group of authors (19).

Teachers were encouraged to interact with caregivers throughout the intervention by communicating with families in-person or *via* phone at least two times a month. However, these interactions were not a requisite part of the intervention or formally structured. Teachers did not receive formal training on caregiver engagement or follow modules specifically targeted toward family engagement.

Teachers and caregivers filled out pre-intervention quantitative measures (“PRE”) in May 2019. Then, teachers delivered targeted, evidence-based mental health interventions to selected students from May to December 2019. During this time frame, teachers received monthly visits from supervising psychiatric social workers to troubleshoot and adapt intervention plans as needed. Teachers and caregivers filled out the same quantitative measures at the conclusion of the intervention in December 2019 (“POST”), creating a 7-month gap between PRE and POST measures.

### Measures

#### Teacher Report Form

The Achenbach System of Empirically Based Assessment (ASEBA) Teacher Report Form (TRF) is considered a “gold standard” for assessing mental health challenges as reported by teachers (30). Teachers complete the TRF based on their perceptions of a student's mental health. The form includes 113 questions that are scored to produce several clinical scores, with the following analyzed for this study: Total Problem score, Internalizing problems, and Externalizing problems. The measure also reports on 14 subdomains (8 empirically validated syndromes and 6 DSM–oriented scales). Raw scores are summed and converted into T-scores. Total problem T-scores <60 are classified as “normal”, 60–63 as “borderline”, and scores above 63 as “clinical”. “Borderline” is defined by TRF authors as likely having symptoms that meet diagnostic criteria for a disorder but with recommended further professional evaluation to minimize false positive diagnoses, where “clinical” is defined as more definitively meeting diagnostic criteria for a disorder (30). Internalizing and Externalizing problem and subdomain T-scores <65 are considered “normal”, 65–69 considered “borderline”, and 70 or greater considered “clinical”. The form is available in Nepali and took participants 20 min to complete.

The tool has been validated globally, including in India, though studies indicate that lower borderline and clinical thresholds may be appropriate for the Indian context (31). TRF authors' recommendations for score thresholds were used as studies have not tested the validity of lower thresholds in the Indian context (31). Still, given concerns about TRF thresholds for the Darjeeling context and that “borderline” scores indicate likely having symptoms that meet diagnostic criteria for a disorder (as above), we consider the borderline cutoff for all TRF scores to be the threshold above which a child meets criteria for a disorder and needs indicated care.

For each student, two teachers each filled out the TRF PRE and POST to decrease rater bias. Limited resources precluded data collection by an independent observer. The teacher delivering care to the assessed student was the “primary observer”. A second enrolled teacher in the school who knew the assessed student well and who was not delivering care to the assessed student was the “secondary observer”.

#### Strengths and Difficulties Questionnaire

Caregivers assessed their child's mental health through the Strengths and Difficulties Questionnaire (SDQ) (32). The SDQ is a standardized and validated self-administered form that screens for mental health struggles among children ages 4–16 years over the previous 6 months. As a newer instrument, it has not been validated for the Indian or Nepali contexts; however, it has been used by several studies in India for its global scoring system and brevity and has been shown to have acceptable reliability in one study in western Nepal, with a Cronbach's alpha of 0.69 (33, 34). Twenty-five items are rated on a 3-point scale (0–2), with 0 = not true and 2 = certainly true, with 5 items reverse scored. Raw scores are summed and grouped to produce scores for: Total difficulties (14–16 considered “borderline”; 17–40 “abnormal”), Externalizing difficulties (8–10 considered “borderline”; ≥11 considered “abnormal”), Internalizing Difficulties (6–8 considered “borderline”; ≥9 considered “abnormal”), and Impact of mental health on one's life (greater than ≥1 was considered to “have impact”) (35). The SDQ was collected PRE and POST. The form is available in Nepali and typically took participants 10 min to complete.

#### Annual Status of Education Report

Widely used in India since 2005, the Annual Status of Education Report (ASER) is a rapid assessment of a child's reading and math capabilities that is validated and reliable in the rural Indian context (36). Given the wide range of the quality of education and family literacy across India (36), the age range covered by the ASER is wide (ages 5–16 years) despite low maximum grade levels achievable on the test (Grade 2 in reading, Grade 4 in Math). To assess student academic achievement with granularity, we utilized the extended ASER assessment with 4 reading questions assessing word and sentence meaning and 7 math questions assessing a math level ranging from number recognition to word problem completion. A 1-point difference in either score is considered meaningful as it indicates the presence of an additional skill. A trained member of the research staff administered the ASER orally to each child in Nepali using the Nepali version, requiring 10 min per child.

### Analysis

Demographics of teachers, caregivers, and children were descriptively explored. Demographics of teachers who completed all study activities were compared to those of teachers who dropped out of the study to determine whether there were significant group differences. All data were cleaned and reviewed for completeness prior to the analysis. There were data missing for two children PRE and POST on the TRF, while there was data missing for one child PRE and POST on the SDQ. Missing data were investigated in collaboration with the field team to ensure that the dataset was as complete as possible. Children who still had data missing following this investigation were removed from the corresponding analyses. For demographic data, independent sample *t*-tests were used when comparing continuous variables, while Fisher's exact tests were used for categorical variables.

Mean scores were calculated at PRE and POST time points for the TRF (Total Problem, Externalizing, and Internalizing scores), SDQ (Total Difficulties, Externalizing Difficulties, Internalizing Difficulties, and Impact scores), and ASER (Math and Reading). Paired sample *t*-tests (two-tailed) were used to compare means PRE to POST. Due to the preliminary nature of the analysis and the study's small sample size, paired sample *t*-tests were used rather than regression analyses. The sample was not adequately powered for multivariable analysis. Because the results of a multivariable analysis would likely be extremely underpowered, *t*-tests were employed instead to better represent the exploratory results. Likewise, potentially confounding variables were not controlled for in the analysis as the small sample size would likely lead to an unstable model producing unreliable results.

## Results

Descriptive statistics were obtained for all participant demographics (Tables 2A–2D). Teachers who completed all study activities (*n* = 13) were typically young (mean age of 29.85) and female (76.9%). The group was split in terms of education. Five teachers attended primary or secondary school, but not undergraduate; eight teachers obtained some undergraduate education or more; five had formal training to become a teacher; and six obtained a teaching certificate (Table 2A). Children targeted for intervention were mostly male (69%) and displayed a variety of mental health concerns (externalizing: 20%; internalizing: 52% at program enrollment; Tables 2B, 2C). Caregivers of participating children were mostly mothers (62.1%), with a few fathers, aunts, grandparents, and non-biological guardians included in the group as well (Table 2D).

**Table 2A:** Teacher demographics and comparisons of teachers who did and did not complete all study activities.

**Variable**	**Enrolled** ***N* = 19**	**Comparison of teachers who did and did not complete all study activities**		
		**Completed** ***N* = 13**	**Did not complete** ***N* = 6**	***T*-test *p*-value^**∧**^**
**Continuous variable**	**Mean (SD)**	**Mean (SD)**	**Mean (SD)**	
Age	28.4 (5.8)	29.85 (5.9)	25.17 (4.4)	0.10
Years teaching	6.1 (5.4)	7.4 (5.8)	3.33 (3.4)	0.13
Years at current school	4.4 (4.5)	4.96 (5.0)	3.33 (3.4)	0.48
**Categorical variable**	***N*** **(%)**	***N*** **(%)**	***N*** **(%)**	**Fisher's exact test** ***p*****-value**^**∧**^
Female gender	16 (84%)	10 (76.9%)	6 (100%)	0.52
**Language**				
Nepali	8 (42%)	5 (38.5%)	3 (50.0%)	0.99
English	6 (32%)	4 (30.8%)	2 (33.3%)	0.99
Hindi	4 (21%)	2 (15.4%)	2 (33.3%)	0.56
**Level of education**				0.54
Some primary	3 (16%)	2 (15.4%)	1 (16.7%)	
Some senior secondary	2 (11%)	2 (15.4%)	0 (0%)	
Finished senior secondary	4 (21%)	1 (7.7%)	3 (50.0%)	
Some undergraduate	4 (21%)	3 (23.1%)	1 (16.7%)	
Finished undergraduate	2 (11%)	2 (15.4%)	0 (0%)	
Some graduate/post-graduate	2 (11%)	1 (7.7%)	1 (16.7%)	
Finished graduate/post-graduate	2 (11%)	2 (15.4%)	0 (0%)	
Had formal training	5 (26%)	5 (38.5%)	0 (0%)	0.13
Teaching certificate	7 (37%)	6 (46.2%)	1 (16.7%)	0.33
**Grade level taught**				
Nursery/kindergarten	8 (42%)	6 (46.2%)	2 (33.3%)	0.99
Class I	15 (79%)	10 (76.9%)	5 (83.3%)	0.99
Class II	14 (74%)	10 (76.9%)	4 (66.7%)	0.99
Class III	12 (63%)	8 (61.5%)	4 (66.7%)	0.99
Class IV	12 (63%)	7 (53.9%)	5 (83.3%)	0.33
Class V	5 (26%)	3 (23.1%)	2 (33.3%)	0.99
Class VI+	3 (16%)	3 (23.1%)	0 (0%)	0.52

∧ *Significant if p < 0.05*.

**Table 2B:** Child demographics.

**Variable**	**Enrolled** **(*N* = 26)**
**Continuous variable**	**Mean (SD)**
Age (years)	8.23 (1.7)
Total household members	3.8 (1.5)
TRF total problem score at enrollment^∧∧^^+^	62.7 (8.6)
**Categorical variable**	***N*** **(%)**
Female gender	8 (30.77%)
Scheduled caste/tribe	7 (26.92%)
Nepali	26 (100)%
English	24 (92.31%)
Hindi	3 (11.54%)
Class I	4 (15.38%)
Class II	6 (23.08%)
Class III	9 (34.62%)
Class IV	5 (19.32%)
Class VI+	2 (7.69%)
TRF externalizing at enrollment^**^^+^	5 (20.0%)
TRF internalizing at enrollment^**^^+^	13 (52%)

∧∧ *T-score ≥ 60 is a borderline score, while >63 is a clinical score*.

** *Had at least a of T-score ≥ 65 to meet the cutoff for a borderline score, inclusive of those with scores ≥70 (clinical)*.

+ *Calculated based on n = 25 children who had a completed TRF and complete demographic data at baseline*.

**Table 2C:** Baseline student mental health profile per the ASEBA TRF.

**Symptom category**	**Number of children with positive symptom *T*-Score (*n* = 25)** ^*^ ^∧^ ^+^
**Scale scores^a, b^**	
Total problem	16 (64.0%)
Internalizing problems	13 (52.0%)
Externalizing problems	5 (20.0%)
**Syndrome scale scores** ^**b**^	
Anxious/depressed	10 (40.0%)
Somatic complaints	4 (16.0%)
Thought problems	8 (32.0%)
Attention problems	4 (16.0%)
Rule-breaking behavior	2 (8.0%)
Aggressive behavior	6 (24.0%)
Withdrawn/depressed	13 (52.0%)
Social problems	8 (32.0%)
Any syndrome scale item positive	19 (76.0%)
**Diagnostic and statistical manual (DSM) oriented scales** ^**b**^	
Depressive problems	10 (40.0%)
Anxiety problems	13 (52.0%)
Somatic problems	1 (4.0%)
Attention deficit	2 (8.0%)
Oppositional defiant problems	3 (12.0%)
Conduct problems	4 (16.0%)
Any DSM oriented scale item positive	17 (68.0%)
**Any symptom item positive^a, b^**	
Total problem, internalizing, externalizing, syndrome, or DSM	20 (80.0%)

* *A positive score was defined as a borderline or clinical score as per TRF author guidelines*.

∧ *Children receiving care could have positive scores in more than 1 symptom category*.

+ *25 out of 26 enrolled children had a completed TRF and complete demographic data at baseline*.

a *Scores under 60 were considered normal for Total Problem T-scores*.

b *Scores under 65 were considered normal for all other T scores*.

**Table 2D:** Caregiver demographics.

**Variable**	**Enrolled** **(*N* = 29)**
**Continuous variable**	**Mean (SD)**
Age of caregiver	33.6 (11.38)
**Categorical variable**	***N*** **(%)**
Female caregiver	18 (69.2%)
**Relationship to child**	
Mother	16 (61.5%)
Father	3 (11.5%)
Grandparent	2 (7.7%)
Aunt	2 (7.7%)
Guardian	3 (11.5%)

Students' mental health statuses were examined as the primary intervention outcome (Table 3). Primary observers rated students' mental health as statistically significantly improving on average on the TRF, going from borderline to normal for the Total Problem score [*t*_(23)_ = −5.93, *p* = 0.0001], and improving in normality for both Externalizing [*t*_(23)_ = −2.74, *p* = 0.01] and Internalizing scores [*t*_(23)_ = −5.97, *p* < 0.0001], where the Internalizing score was on average near the normal/borderline cutoff at PRE. Secondary observers' ratings showed similar changes to those of primary observers, but only Externalizing scores statistically significantly improved [*t*_(23)_ = −2.19, *p* = 0.039] while Total Problem [*t*_(23)_ = −2.06, *p* = 0.051] and Internalizing scores [*t*_(23)_ = −1.77, *p* = 0.09] trended toward significance. By contrast, caregivers' ratings showed a statistically significant decrease on average on the SDQ Impact score [*t*_(24)_ = −2.61, *p* = 0.016] but were “abnormal” PRE and POST for Total Difficulties and Internalizing Difficulties and “borderline” PRE and POST in Externalizing Difficulties.

**Table 3:** Mental health and academic secondary outcomes.

	**Outcome scores**	**PRE mean**	**POST mean**	**POST-PRE mean (95% CI)**	***T*-value**	**DF**	***P*-value**
	**Primary observer (*****n*** **=** **24)**						
**TRF** (*n* = 24)^∧^	Total problem	63.04	54.79	−8.25 (−11.13, −5.37)	−5.93	23	0.0001^*^
	Externalizing problem	58.46	54.13	−4.33 (−7.60, −1.06)	−2.74	23	0.0116^*^
	Internalizing problem	64.17	55.00	−9.17 (−12.34, −5.99)	−5.97	23	<0.0001^*^
	**Secondary observer (*****n*** **=** **24)**						
	Total problem	61.17	55.75	−5.42 (−10.86, 0.03)	−2.06	23	0.051
	Externalizing problem	58.92	52.88	−6.04 (−11.75, −0.33)	−2.19	23	0.039^*^
	Internalizing problem	60.63	55.88	−4.75 (−10.31, 0.81)	−1.77	23	0.09
**SDQ** (*n* = 25)^∧∧^	Total difficulties	18.00	20.16	2.16 (−0.11, 4.43)	1.97	24	0.0608
	Externalizing difficulties	8.64	9.32	0.68 (−0.56, 1.92)	1.13	24	0.2677
	Internalizing difficulties	9.36	10.84	1.48 (−0.18, 3.14)	1.84	24	0.0782
	Impact	2.60	0.8	−1.8 (−3.22, −0.38)	−2.61	24	0.0155^*^
**ASER** (*n* = 26)	Math	4.54	5.73	1.19 (0.30, 2.08)	2.75	25	0.01^*^
	Reading	3.31	3.69	0.38 (−0.01, 0.78)	2.00	25	0.057

* *Significant if p < 0.05*.

∧ *Data missing for 2 children PRE and POST*.

∧∧ *Data missing for 1 child PRE and POST. For the the 3 children who had 2 caregivers each enroll, only the caregiver for each who completed qualitative assessment was included in the comparison such that each child only had 1 caregiver included in the comparison. Thus, 25 caregivers of 25 children were included in the comparison*.

Due to the correlation between child mental health and educational outcomes, academic achievement was examined as a secondary intervention outcome. Children appeared to statistically significantly improve in their math skills POST minus PRE on the ASER by >1 point on average [*t*_(25)_ = 2.75, *p* = 0.01], meaningful as it indicates the attainment of an extra skill on average (Table 3). Reading skills gains POST minus PRE (0.38 point) trended toward significance [*t*_(25)_ = 2.00, *p* = 0.057], translating into some children meaningfully mastering another reading skill while others did not (Table 3).

## Discussion

This study sought to examine the association between participation in Tealeaf, a teacher-led, transdiagnostic, non-manualized task-shifted intervention targeting child mental health, and changes in child mental health status and academics. Primary school-based observers saw significant improvements in child mental health outcomes, while secondary observers noted changes in Externalizing, but not Internalizing, behaviors. Caregivers generally reported few improvements in child mental health status. Academically, significant improvements in math were observed, while reading trended toward significance.

Children at baseline had mental health concerns that spanned all of the categories measured by the TRF, and primary observers saw improvements in child mental health symptoms on average across Total Problem, Internalizing, and Externalizing scores. This finding provides preliminary descriptive evidence for the potential improvement of child mental health symptoms when provided with teacher-delivered mental health care in the form of Tealeaf and Ed-MH. More rigorous study design is needed to draw definitive associational conclusions.

Primary and secondary observer differences in Total Problem scores and Internalizing Problem scores may indicate a bias in primary observers to want their children to improve while also viewing their students as struggling more symptomatically. However, it is also possible that the secondary observers were less familiar with the child who was scored, leading to understating the child's struggles (though differences in PRE-POST secondary observer scores trended toward significance). Evidence for this is potentially seen with the difference in Internalizing Problems scores. Secondary observers rated children to have lower levels of internalizing concerns than primary observers, who, as the children's classroom teachers, may be more attuned to a child with internalizing issues than a passive observer would be. Meanwhile, secondary observers did notice a significant improvement in Externalizing Problems, indicating improvements in challenging behaviors that would likely be more visible to an objective second-hand observer.

It is important to note here that the interpretation of the severity of students' symptoms from either primary or secondary observers' scores should take into account that TRF thresholds for “borderline” and “clinical” are thought to likely be too high for the Indian context (31). Thus, our conservatively set threshold of “borderline” for screening positive may still be set too high. While differences between primary and secondary observers' scores may exist, both sets of scores can be interpreted as children who screened positive or are near positive for having clinical levels of mental health challenges improved or trended toward improvement after receiving mental health care from their teachers.

Caregiver findings are in line with a 2015 review of parent participation and engagement in child mental health treatment in HICs that showed, across 7 studies, parent participation being correlated with improved functioning and impairment but mixed improvement in child mental health statuses (37). Studies in this review included varying levels of parent engagement, which may underlie the mixed mental health outcomes reported. Specifically, in the context of the present study, teachers may have provided mental health care that led to a visible impact in the school setting but that did not translate to the home due to a lack of caregiver implementation of program tools. Prior research examining family engagement in a separate trial of Tealeaf indicated that many caregivers lacked a developed understanding of the program and of child mental health, limiting caregiver use of Tealeaf techniques in the home (38). Thus, it is possible that more formal and active caregiver involvement in Tealeaf could lead to more noticeable improvements in child mental health status in the home setting, leading to improved future parent ratings.

Improvement in math skills on the ASER may be related to improved mental health as mental ill health has been linked with poorer academic outcomes (39). However, improved academic achievement was likely multifactorial, including the natural progression children may have made by receiving an education regardless of having received care. Still, that children were not documented to fall behind academically may indicate a partial role mental health care may have played in improved academic achievement; other studies have documented children's mental health struggles contributing to their falling behind academically (40, 41). In addition, while math skills significantly improved following the intervention, reading skills did not. This discrepancy suggests that for the targeted students, school attendance did not guarantee significant academic improvements across all domains; otherwise, reading skills might have improved at the same rate as math skills. These results suggest that additional factors, potentially including activities associated with Tealeaf, may have been at play. Finally, qualitative results of separate manuscripts indicated that many teachers attributed academic improvements among targeted students to Tealeaf (19); Cruz et al.^1^. Some teachers reported spending a greater amount of one-on-one time with targeted students, engaging both in therapeutic activities and additional academic support during those sessions. These qualitative data provide insights into the potential role Tealeaf may have played in supporting student math achievement, as teachers reported extra efforts to bolster both child mental health and academics in individual sessions through the program. Overall, while a multitude of factors undoubtedly contributed to the observed improvements in math skills, the significant changes in math, but not reading, along with the qualitative reports of teachers providing additional one-on-one academic help for targeted students during relationship-building sessions, suggest that Tealeaf activities may have had some influence on these academic gains.

### Implications and Future Directions

While preliminary and limited, these results indicate the potential association of participation in the Tealeaf intervention with improvements in child mental health status and academics. The results thereby contribute to the evidence base of literature supporting the potential role of teachers as agents of mental health care delivery for students in their classrooms. In addition, these results add credence to the potential emergence of Ed-MH as a new modality of care provision. Unlike many manualized, school-based interventions, Tealeaf uses Ed-MH, empowering teachers to (1) selectively choose evidence-based techniques suitable for the targeted child through the creation of individualized intervention plans; (2) adapt those plans as needed throughout the course of intervention, troubleshooting challenges as they arise; and (3) deliver mental health intervention through a dynamic and flexible approach, interspersing academic learning with the delivery of mental health care consistently throughout the course of a 6-hour school day. Further, Tealeaf is transdiagnostic, allowing teachers to work with any child with any mental health struggle. Having the Tealeaf and Ed-MH therapeutic skill set allows teachers to have a reach more similar to a mental health professional than a lay counselor trained in manualized, diagnostic-specific care.

Results of the current study indicated that children participating in the program improved in mental health symptoms. Accordingly, these results support that teacher-led interventions that follow the structure of Ed-MH and Tealeaf can potentially lead to improved child mental health outcomes. Teachers did report challenges during intervention implementation, including logistical barriers (such as finding time to meet individually with the student), difficulties for some with the implementation of specific program tools (such as use of the AABC Chart), and difficulties engaging families (such as scheduling meetings between teachers and families), discussed in a separate manuscript (19). Still, as preliminary results, the findings presented here are a potentially impactful contribution to the literature. These findings, in conjunction with findings from other manuscripts from this group of authors, demonstrate how Tealeaf may overcome several systemic challenges in implementing school-based mental health as delineated by Gee and colleagues: (1) quality of therapy training, (2) a lack of time of school professionals to deliver the intervention, (3) coordinating mental health care with academic scheduling, (4) community stigma, (5) contradicting priorities of health and education systems, and (6) a lack of support from families (12). In other manuscripts, this group of authors has demonstrated that (1) Tealeaf training results in improved teacher knowledge and increasingly psychologically-minded teacher behavior (18); (2) that most teachers find the time commitment to deliver this form of care feasible (19); (3) that teachers are able to coordinate delivery of care with academic scheduling (19); (4) that the structure and implementation of Tealeaf may allow it to be a community's first step toward addressing stigma (19); (5) that it can align health and education priorities in one intervention (19); and (6) that families and teachers find family engagement crucial in Tealeaf, leading this group to develop a formal family component to Tealeaf (38). The preliminary results from this manuscript indicate the potential of Tealeaf to foster improvements in child mental health symptoms, while results from other manuscripts from this group of authors point to the ability of Tealeaf to overcome several common barriers in the delivery of school-based mental health care. Together, these promising results support further investigation of Tealeaf in a powered study with a more rigorous design to determine whether children's mental health symptoms improve after receiving indicated levels of task-shifted mental health care from their teachers. A powered trial is planned to commence in 2022.

Notably, however, child mental health symptoms did not improve as reported by families, implying that symptom improvements did not translate to the home environment. In concert with findings from other manuscripts indicating the value of caregiver involvement in Tealeaf (38); Cruz et al.^1^, as well as a robust literature demonstrating the importance of caregiver participation in their children's mental health care (37, 42), this finding further supports the need to formally involve caregivers in Tealeaf. A formal caregiver component is currently being developed.

While academic outcomes are the result of multiple factors, discussed previously, results here do indicate the potential for Ed-MH and Tealeaf to play a role in improving math outcomes. As mental ill health has been well-studied to negatively affect children's academic achievement (41), a powered study to determine whether children's academic achievement improves after receiving Ed-MH care in Tealeaf is warranted. Such a study should carefully measure covariates likely to impact academic achievement. The findings of such a study could be of great interest to school districts and Departments or Ministries of Education who are often needing to support children with poor mental health and, if findings were positive, could do so efficiently with Tealeaf (9).

### Limitations

The results of this analysis come from a small, pilot trial using pre-post methods and are accordingly limited by the small sample size and lack of a comparison group. Although statistical tests least sensitive to small sample size were used, the use of a small sample still could have potentially led to skewed results. Due to small sample size, statistical analyses also did not account for the nesting of students within schools, which may have inflated the significance of the results. In addition, the size of the trial limited the demographic diversity of participants, and results may not generalize to the greater Darjeeling area or other LMIC regions. Potential biases in TRF data collection for primary observers and attempts to mitigate bias with secondary observers with less than ideal knowledge of the rated children may have also influenced results. Rather than independent evaluators, teachers with knowledge of the children rated child behavior and mental health. While teachers likely hold the greatest understanding of each individual child given their consistent access to students, perhaps increasing the accuracy of their responses, this familiarity could also potentially lead to biased ratings due to their personal relationships with the individual children. This subjectivity, which would not exist with the use of an independent rater, potentially served as another study limitation.

## Conclusions

The current study examined preliminary evidence for the ability of a teacher-led, task-shifted intervention implemented in an LMIC to foster significant improvements in child mental health and academic outcomes. Results suggested that this type of intervention that is transdiagnostic and non-manualized may potentially significantly improve child mental health outcomes and may play a role in improving educational outcomes. These results thus hint at the viability of teacher-led, school-based interventions, specifically Tealeaf and Ed-MH, as a method of significantly improving child outcomes and reducing the global child mental health care gap. Future directions include building on the results of this work through the implementation of a larger, randomized-controlled trial of Tealeaf to determine whether these results can be replicated on a larger-scale. In addition, future directions should include further feasibility testing of Tealeaf and Ed-MH to troubleshoot potential barriers related to time, scheduling, and caregiver involvement. Methods of overcoming additional systemic barriers to school-based mental health intervention, such as community mental health stigma, should be explored as well, potentially through novel interventions designed to engage families and community members in child mental health. If these barriers are overcome and more rigorous testing reveals continued significant results, the implementation of Tealeaf and other Ed-MH-based interventions could reduce the child mental health care gap and improve outcomes for children globally.

## Data Availability Statement

The datasets presented in this article are not readily available because the datasets generated and/or analyzed during the current study are not publicly available for ethical reasons. Requests to access the datasets should be directed to christina_cruz@med.unc.edu.

## Ethics Statement

The studies involving human participants were reviewed and approved by University of North Carolina Institutional Review Board and Darjeeling-Based Ethics Committee. Written informed consent to participate in this study was provided by the participant or the participants' legal guardian/next of kin.

## Author Contributions

CC, KH, and MM designed the study. BG was involved in study design. PG, SB, and AT delivered the teacher training, provided supervision, and collected data. CD collected data. CC and MM provided supervision to PG, SB, and AT as they provided supervision to the teachers. AR and ML performed data analysis. JV, CC, ML, AR, PG, SB, and MM were involved in data interpretation. JV, CD, and CC drafted the manuscript. All authors revised and approved the final version of the manuscript before submission.

## Funding

The results in this publication regarding data collected from caregivers and students were made possible through the American Academy of Child and Adolescent Psychiatry (AACAP) Junior Investigator Award, supported by Pfizer and Sunovion Pharmaceuticals; its contents are the responsibility of the authors and do not necessarily reflect the official views of AACAP nor the companies listed above. The results in this publication regarding data collected from teachers were made possible through an Early Career Award from the Thrasher Research Fund; its contents are the responsibility of the authors and do not necessarily reflect the official views of the Thrasher Research Fund. The publishing of this manuscript is made possible by support from the Doris Duke Charitable Foundation through a Caregivers at Carolina Award from the Fund to Retain Clinical Scientists; the manuscript's contents are the responsibility of the authors and do not necessarily reflect the official views of the Doris Duke Charitable Foundation.

## Conflict of Interest

CC, PG, and MM hold the copyright to the training and intervention materials for the teacher-led task-shifted alternative system of children's mental health care that this research studies. The remaining authors declare that the research was conducted in the absence of any commercial or financial relationships that could be construed as a potential conflict of interest.

## Publisher's Note

All claims expressed in this article are solely those of the authors and do not necessarily represent those of their affiliated organizations, or those of the publisher, the editors and the reviewers. Any product that may be evaluated in this article, or claim that may be made by its manufacturer, is not guaranteed or endorsed by the publisher.

## Footnotes

^1^Cruz CM, Dukpa C, Vanderburg J, Rauniyard A, Giri P, Bhattarai S, et al. *Acceptability and Exploration of Child Outcomes of Teacher-Delivered Task-Shifted Child Mental Health Care to Teachers, Caregivers, and Students in a Low and Middle Income Country: A Mixed Methods Pragmatic Pilot Study*. Under review.
